# Reasons for early marriage of women in Zahedan, Iran: a qualitative study

**DOI:** 10.1186/s12905-022-02127-9

**Published:** 2022-12-23

**Authors:** Maryam Hosseini, Khadijeh Asadisarvestani

**Affiliations:** 1 Department of Social Sciences, Faculty of Humanities, University of Bojnord, Bojnord, Iran; 2 Department of Demography and Geodemography, Faculty of science, Charles University, Czechia Prague, Czech Republic; 3 Department of Social Sciences, Faculty of Literature and Humanities, University of Sistan and Baluchestan, Zahedan, Iran

**Keywords:** Early marriage, Girls, Sistan and Baluchestan, Qualitative content analysis

## Abstract

**Background:**

Early marriage occurs in some regions of the world. Given that early marriage can have many negative consequences for girls by affecting their physical, mental, and social health, the purpose of this study was to investigate the reasons for early marriage from the perspective of women who had experienced it in Zahedan, Iran.

**Methods:**

This qualitative research was conducted based on the qualitative content analysis method in 2020–2021. The target population included women aged 18–35 living in Zahedan who were married under the age of 18 years. Purposeful sampling was used to select the participants. In order to collect data, in-depth interviews were performed to decipher the reasons for early marriage from these women’s perspectives. A total of 36 interviews were conducted from October to December 2020 until reaching theoretical saturation. Conventional content analysis was used to analyze the data and extract the relevant concepts and categories.

**Results:**

The analysis of the interviews in this study revealed three main categories:(1) “early marriage as a transcendental coercion” ( early marriage as a cultural heritage and early marriage as a control tool, girls’ weaknesses and inability to resist, dominant attitudes toward gender and gender restrictions), (2) “early marriage as a solution or a kind of problem solving” (escape from undesirable family environment, escape from financial poverty, and strategic escape from an unpleasant situation), and (3) “early marriage as a voluntary act” (real agency and imaginary agency). To substantiate these findings, we presented excerpts from the interviews conducted with the participants.

**Conclusion:**

The results of this study suggest a multidimensional picture of early marriage. It seems that improving cultural conditions and empowering families and girls in various areas, including marriage, can reduce the rate of early marriage and/or at least mitigate its undesirable consequences.

## Background

Marriage is one of the most important events in human life [1]. The quality, manner, and durability of marriage as well as satisfaction with it are closely related to social changes and the prevailing philosophical attitudes in the society [2, 3]. In this regard, one of the issues that need to be discussed in various scientific fields and institutions is the age of marriage, which has legal, psychological, moral, social, and educational dimensions [4].

The age of the first marriage is defined according to the cultural, social, religious, and ethnic specifications of each society [5–7]. In the traditional model of marriage, parents play a major role in the age of the first marriage as well as the choice of the child’s partner [8].

The age of marriage, both legally and culturally, varies from country to country, and there are many debates supporting marriage at an older age or a younger age [9]. Although most girls today marry at an older age, early marriage is still common in many countries like Pakistan [9], Bangladesh [10], Malawi [11], Ethiopia [12], and Nepal [13]. Generally, this phenomenon occurs especially in developing countries and the regions where out-of-wedlock childbearing is not approved [9, 14, 15]. In these regions, the age of the first marriage is influenced by a range of social, economic, and cultural factors (such as ethnicity and education level), especially in women [9].

According to the United Nations International Children’s Emergency Fund (UNICEF) definitions, early (or child) marriage denotes formal marriage or informal union before the age of 18 [16]. On the other hand, according to Part 1, Article 1 of the Convention on the Rights of the Child, “a child means every human being below the age of eighteen years unless under the law applicable to the child, majority is attained earlier”. From a legal perspective, early marriage equals to child marriage, meaning that the spouse or one of the couples is a child, and the marriage takes place under civil, religious, or customary laws, with or without official registration. Girls under the age of 18 years are physically, physiologically, and psychologically unprepared to take responsibility for marriage or having children. Likewise, it is difficult for boys of the same age to take responsibility and provide for an independent life [16, 17].

Since most countries define a legal age for marriage, marriages occurring under legal age are either not officially registered or poorly registered. In addition, marriage and divorce registration, particularly in low-income countries, is the most unreliable and unavailable service in relation to vital events [18]. Poor registration of marriage can be due to various reasons, including unpopular type of marriages (e.g., polygamy, temporary marriage, or concealed marriage), prohibition of marriage under legal age, and great distance from a registration office, particularly for people living in rural areas [1, 19, 20]. Therefore, the available statistics cannot be accurately cited. It is estimated that 130 million girls will have early marriages between 2010 and 2030 (i.e., 14 million per year), and many will be under the age of 15. Also, more than 700 million women are currently married children, half of whom reside in South Asia [21]. According to the latest statistics released by the UNICEF, the prevalence of child marriage for children under the age of 15 is 5% worldwide and 3% in Iran. In 2020, a fifth (20%) of the children worldwide and 17% in Iran were married under 18 years of age [22].

It is documented that early marriage can have many negative consequences for girls, impairing their physical, mental, and social health. Psychologically, early marriage is associated with an increased incidence of mood disorders such as depression, anxiety, obsessive-compulsive disorder, and even suicide [23]. From the social perspective, early marriage can lead to early divorce, marital conflicts, spousal abuse, domestic violence, running away from home, infidelity and extramarital affairs, spousal homicide, illiteracy, insufficient development of social and marital skills, and finally social isolation [24–35]. Marriage at a young age can also increase physical and sexual problems [36] and even sexual violence and forced sex [14, 37]. In addition, early pregnancy and preterm delivery, increased mortality of pregnant adolescents during labor, increased HIV and other sexually transmitted infections, higher neonatal mortality and anemia, exaggerated risk of preeclampsia and postpartum hemorrhage, and complicated and prolonged labor are among other consequences of early marriage [38–43].

According to Article 1041 of the Civil Law of the Islamic Republic of Iran, the legal ages of marriage are 13 and 15 years for girls and boys, respectively. In the same article, it has been noted that the marriage of girls or boys before the legal age requires the permission of the father and a competent court that will decide on whether or not expediency is observed, thus providing a legal justification for early marriage.

Although child marriage is not common in Iran, it exists in less developed places and among specific cultures and ethnicities, where it is an accepted social and cultural norm. Contrary to the general belief that early marriages are prevalent in rural and tribal areas, early marriage takes place at a higher rate in some urban areas, especially those that have remained more conservative and traditional and/or have retained a strong sense of ethnic identity [1]. Kian-Th ebaut’s study of various ethnic and linguistic groups In Iran estimates the average rate of early marriage at 16%, with Sistan and Baluchestan province having the highest rate [44]. However, few studies have been conducted on early marriage in Iran, particularly Sistan and Baluchestan province. Additionally most of the previous studies have dealt with this issue from demographic or health perspectives.

Considering the above discussion, the present qualitative study seeks to clarify the reasons for early marriages in Zahedan as well as to investigate the perception of women who marry under the age of 18. The findings of this research can provide a deeper and more realistic understanding of early marriage because it relies on the voices and perceptions of women who are actually involved in this phenomenon.

## Methods

### Study design

This research is designed using a qualitative approach based on conventional content analysis. Qualitative methods allow the researcher to get a deeper understanding of the lived experience of the participants and their perception of the world.

### Study settings

The study participants consisted of married women who lived in Zahedan, Sistan and Baluchestan, Iran. Purposive sampling was used to select the participants. The inclusion criteria were women aged 18 to 35 years, living in Zahedan, having the experience of marriage under the age of 18, and willingness to participate in the study. On the other hand reluctance to do the interview and lack of favorable conditions to continue the interview were the exclusion criteria.

### Data collection

In this research, the required information was collected through in-depth interviews to examine the reasons for early marriage from the perspective of women who had married under the age of 18. The interviews were conducted from October to December, 2020. The interviews lasted between 45 and 100 min and were performed in a semi-structured manner. The participants freely answered the questions raised during the interview and talked about the reasons for early marriage. The participants could choose whether to have the interview audio-taped or not. For this reason, some of the interviews were recorded with an audio recorder and the rest were taken notes of and then analyzed. The researchers continued the interviews until reaching theoretical saturation. Overall, 36 interviews were conducted.

### Data analysis

This qualitative research was conducted based on qualitative content analysis. According to Hsieh and Shannon, the existing approaches to content analysis can be divided into three categories: (1) conventional content analysis, (2) summative content analysis, and (3) directed content analysis [45]. Considering the aim of the present study and the problem that is to be addressed here, we adopted the conventional content analysis. This method is commonly used when there are limited theories and literature available on the phenomenon under study.

Content analysis started after the first interview. The interviews were examined several times to gain a full understanding of their content. Accordingly, the initial analysis was conducted to identify the primary codes. The codes were then categorized, based on their similarities and differences, and organized under meaningful clusters. Depending on the quality of the relationship between the subcategories, we merged and organized these subcategories under the main categories. In the next step, a definition was provided for each category, subcategory, and code.

Without any theoretical background, encoding and categorization of the data through conventional content analysis began simultaneously with the examination of the interviews. Depending on the subject, meaning units were determined step by step and then compressed to determine codes. In case of the presence of a common background, the codes were merged to determine the categories. Finally, a general concept, resulting from the combination of these categories (themes), was obtained.

### Reliability

Validity and reliability in qualitative research need to be confirmed from the perspectives of the reader, participant, and researcher [45, 46]. According to Creswell, qualitative researchers should use at least two strategies to evaluate the validity of their research. In this study, we used three strategies: thick description, peer review, and cross-checking by the participants [46].

Two experts in qualitative research reviewed the results and judged their validity. In this process, a number of categories and concepts were reassessed. For participant validation, the concepts and categories extracted from the interviews were presented to a number of interviewees, and they were asked to evaluate the themes and categories and comment on their validity. The reliability of our collected data was also approved by the participants. In order to increase data richness, we analyzed and interpreted all the concepts and categories in detail and substantiated them by excerpts from the interviews.

### Ethical considerations

The ethical considerations were observed in this study. At the beginning of each interview, the researcher introduced herself, provided the necessary information to the participant, and acquired her informed consent. During the interview, it was tried to answer any ambiguities and questions raised by the participant. The place of the interview was chosen according to where the participant felt comfortable. In this study, we used pseudonyms for the subjects, and the information they did not want to be disclosed remained confidential.

The protocol of this study was approved by the Research Ethics Committee of University of Sistan and Baluchestan (code: IR.USB.REC.1399.013) and it confirmed that all methods were carried out in accordance with relevant guidelines. The participants signed a written informed consent form. There was no illiterate participant nor any subject was less than 16 years old. All procedures of this research have been performed in accordance with the ethical standards of the Helsinki Declaration.

## Results

### Participants’ characteristics

Overall, 36 married women aged 18 to 35 years (mean age of 24), who lived in Zahedan and had married under the age of 18, were enrolled. The mean age of first marriage among the participants was 16 years (the range was 12–18 years). Notably, 15 participants continued their education after marriage, and 21 others were housewives. Regarding ethnicity, 10 were Fars and 26 were Baluch. The participants’ husbands were in the age range of 21 to 48 years (mean age was 35 years); 23 of them had diplomas or lower degrees, and 13 had a university degree. Some of the socio-demographic characteristics of the participants are listed in Table 1.

**Table 1:** Socio-demographic characteristics of the participants

Age group	18–20	8
	21–23	7
	24–26	10
	27–29	5
	30–32	3
	33–35	3
Mean age	24	
Mean age at first marriage	16	
Educational level	Illiterate	0
	Primary school	8
	Secondary school	15
	College and higher education	13
Ethnicity	Fars	10
	Baluch	25

Analyzing the interviews suggested that women’s reasons for early marriage could be organized in nine subcategories placed in three main categories (Table 2): (1) Early marriage as a transcendental coercion (consisting of early marriage as a cultural heritage and a control tool, girls’ weaknesses and inability to resist, dominant attitudes toward gender and sexual restrictions); (2) Early marriage as a solution or a type of problem-solving (consisting of escape from undesirable family environment and financial poverty, and strategic escape from an unpleasant situation); and (3) Early marriage as a voluntary act (consisting of real agency and imaginary agency). Below these factors are fully discussed along with direct evidence from the interviews.

**Table 2:** The Themes, main categories and subcategories on the reasons for early marriage

Them	Main category	Sub-category
Reasons for early marriage	(1) Early marriage as a transcendental coercion (extra-personal coercion)	1-1-Early marriage as a cultural heritage 1-2-Early marriage as a control tool 1-3-Girls’ weaknesses and inability to resist 1-4-Dominant attitudes toward gender and gender restrictions
	(2) Early marriage as a solution or a type of problem-solving	2-1-Escape from undesirable family environment 2-2-Escape from financial poverty 2-3-Strategic escape from an unpleasant situation
	(3) Early marriage as a voluntary act	3-1-Real agency 3-2-Imaginary agency

### 1. Early marriage as a transcendental coercion

Transcendental coercion(extra-personal coercion) includes social pressures outside the personal domain that are rooted in cultural rites and traditions, family and ethnic norms, and gender attitudes. Under this category, there were women who, for various reasons, emphasized compulsion in their early marriage and the fact that they were passive and had no role in the process.

### 1-1-Early marriage as a cultural heritage

Evidence shows that in the city of Zahedan, marriage at an early age is considered a normal behavior and is culturally supported by local customs. Some of the girls who married at a young age mentioned that early marriage was customary in their place of residence. For instance, one of the interviewees mentioned:

*“In my father’s family, it is customary to get married early, and according to the custom, I should have gotten married early. In their opinion, marriage at a young age is much better“(female, 25 years old).*

Another interviewee referred to a custom named “Naf Boran”, during which a girl and a boy are married in childhood and even in the fetal period. Their elders make agreements between themselves, and breaking these promises by one of the parties is culturally unacceptable. In this regard, a participant mentioned:

*“Our marriage was a consanguineous marriage. The elders, like my grandfather, had already decided about us” (female, 30 years old).*

### 1-2- early marriage as a control tool

It seems that some families use early marriage as a tool to control their girls. They sometimes think that if a girl gets older, it may be difficult to control her or resist their wishes for marriage, so they prefer Escape from undesirable family environment to arrange her marriage at a younger age. On the other hand, sometimes they arrange early and planned marriages to punish girls and curb their impudence. For example, one of the interviewees observed:

*“I was young at the time, and I had a stranger suitor that I loved very much, but my family forced me to marry one of our relatives to forget the person I loved. I went to the brink of suicide, but they did not concede. I hate my family for this marriage” (female, 31 years old).*

### 1-3- girls’ weaknesses and inability to resist

Another reason for early marriage in this area under study is the inability of girls to achieve their dreams. Due to their inability to oppose their families’ demands, women and girls tend to passively submit to arranged marriages in which they have no role. For example, one of the participants noted:

*I was not aware of my family’s decision to accept my husband’s proposal. After I realized, I had to get married to protect my family’s reputation, because in our region, if the girl refuses the decision in this condition, it is a kind of disgrace (female, 28 years old).*

Another participant described her passivity thus:

*“I had no role; I was deprived of the right to choose. Without prior notice and without my knowledge, I was forced by my father to come to the marriage* ceremony*” (female, 35 years old).*

This passivity and lack of perseverance persistence sometimes leads to marriages in which there is no feeling of satisfaction and love, and the spouse is forced to live with bitterness for the rest of their life. This feeling failed resistance can both endanger one’s mental health and lead to domestic and possibly sexual violence. For example, a participant affirmed her hatred of her husband, and revealed that she had even thought of murdering him or committing suicide:

*“I hate my husband, and I want to divorce him, but my family is an obstacle, and if I could, I would kill him. I’m psychologically and mentally disoriented and even think of suicide as the only solution*” *(female, 23 years old).*

In areas where forced and early marriages are customary, women are deprived of both the right to choose their husband and the right to end their marriage and get a divorce, a decision in which families have a fundamental and decisive role. The rising rates of suicide, homicide, and domestic violence in some areas could be the result of this approach to marriage.

### 1-4- attitudes toward gender and gender restrictions

In the culture of the region under study, having boys is preferred to girls, hence families’ lesser attention to girls. Culturally, strict familial and gender restrictions for girls and their hope for fulfilling their unmet financial needs and aspirations at the spouse’s home further encourage girls to acquiesce to early marriage and become independent of their parents. Thus, an interviewee acknowledged:

*“I got married early so that I could have my own life, and no one could restrict me anymore, so I could be free. I had a strict family“(female, 21 years old).*

### 2- Early Marriage as a Solution or a Type of Problem-Solving

The analysis of the interviews showed that sometimes accepting and conceding to an early marriage is a personal decision, but this decision is made in response to the existing conditions and serves as a problem-solving technique. These conditions are described below.

### 2-1-escape from undesirable family environment

Some individuals who willingly accept early marriage are in the midst of family conflicts and have experienced lack of family support, dysfunctional relationships and weak emotional bonds between family members, and other similar problems. Therefore, in order to replace this condition with a desired one, they decide to get married early. For example, one of the participants declared:

*“My parents had fights at home every day, and there were a lot of family disputes, and I decided to get married for my own comfort and to be away from home” (female, 24 years old).* Another participant mentioned:

*“I did not have a good family, my family was indifferent to me and constantly berated me, and I could not stand the indifference, so I had to get married” (female, 22 years old).*

### 2-2- escape from financial poverty

Poverty in the family is another reason for girls to get married early. Marriage and starting an independent life can sometimes free a person from the family’s poverty. Therefore, in this case marriage can seem a rational decision to escape from poverty and achieve a desired situation. In this condition, marriage reduces the financial burden on the father, but at the cost of a daughter’s life. One of the participants said:

*“We did not have a good life. My family was not in a good financial state. I wanted to have an ideal life to meet all my needs” (female, 27 years old).*

Another participant revealed:

*“My family did not have a good financial situation, my father could not afford the costs, so I had to get married early (female, 21 years old).*

### 2-3-strategic escape from an unpleasant situation

Deciding to get married early can sometimes be a wise decision to escape from an unpleasant situation. Since in the cultural context of this region, girls do not have much power to resist the coercion of elders, this strategic decision can save them from such an unfavorable and unsafe environment. One of the participants noted:

*“I loved my cousin, but at the same time, I had another suitor, and because he was a good man, my family was at a crossroads. He did not want to give up, so I was afraid my family would accept his proposal, so I got married to my cousin as soon as possible so they would leave me alone” (female, 22 years old).*

Another woman said:

*“Since I was a child, it had been arranged that I should marry a particular man, but I did not like him. When my husband proposed to me, I accepted because he had a better situation, and we got married quickly so that I would not have to marry the other person (female, 33 years old).*

On the other hand, rejecting a marriage proposal can occasionally end up in various forms of severe violence against girls [47], so early marriage in this condition can be a strategic and reasonable choice to stay safe.

### 3- Early marriage as a voluntary act

In voluntary acts, a person consciously and freely makes a decision and acts on it. Some of the interviewees pointed out that their early marriage was voluntary and they were satisfied with it. However, the analysis of their interviews showed that these people can be categorized into two groups. First, those who decided to get married at a young age as a result of studying and acquiring sound knowledge (hence real agency); second, those who did not have the necessary skills to choose their spouse and their decision is based on emotions (hence imaginary agency).

### 3-1-real agency

*A small* number of participants had decided to get married after careful thinking and investigation and based on their own free will. Considering that in other more developed cities of the country, girls at an early age do not properly and sufficiently learn the required spouse-selection skills and even life skills, it is not unexpected that only a small number of girls in Sistan and Baluchestan region could obtain such skills at this age. Some participants talked about their consent and willingness to marry at an early age. For example, one of the participants expressed:

*“I had a suitor who had a reputable family, and he fulfilled the criteria for being a good husband. On the other hand, I loved him, so I agreed to marry him. I did not want to lose him” (female, 20 years old).*

Similarly, an interviewee observed:

*“I already knew my suitor, and there was an interest between us, so after he proposed to me, we decided to get married sooner” (female, 25 years old).*

Another participant referred to her careful thinking about the criteria of choosing a spouse:

*“My spouse had the criteria that I had in mind, and we had the same faith and morals, and he was a good man, so I accepted his proposal“(female, 20 years old).*

### 3-2-imaginary agency

In this condition, a person thinks that the decision of getting married is based on their own free will, but a deeper assessment of their statements show that due to the lack of spouse-selection skills, their free will and personal decision have been overshadowed by emotions and people’s words. Thus, the decision has not been based on conscious agency. For example, one participant said:

*“I did not have to get married, but I made an irrational and emotional decision while I thought it was a good decision“(female, 22 years old).*

Another participant said:

*“My mother and brother said that he is a good suitor, and the fact that interest emerges after marriage. So, I trusted their words, but because of this trust, my life was ruined, and no interest was formed either. I feel hatred; I am careless about my future, and I have no motivation anymore” (female, 26 years old).*

The final point to consider is that a large percentage of these girls, even though they willingly married at a young age, spoke of problematic experiences in their family and personal lives, such that most of them did not want their daughters to get married at a young age. The two main reasons for this attitude are as follows:

1. *Unfulfilled needs and expectations and personal failure* This group of girls believed that many of their dreams were shattered by getting married at a young age. In adolescence and youth, which should be full of energy and happiness, they became engaged in family issues that they were not yet ready to handle. Many did not pursue their education and dreams and now spoke with regret about their desire to continue their education, even though a large proportion of the participants had succeeded to overcome this barrier and continue their education.
2. *Experiencing family problems and conflicts* Marriage at an early age deprives girls of the opportunity to acquire the knowledge and skills necessary for married life, leading to marital problems, conflicts, and even violence. The participants stated that they did not want their daughters to live under coercion and enter into a married life when they knew nothing about it, because, they believed, such a situation will lead to more conflicts and domestic violence. They wished for their daughters’ lives to pass in better circumstances with fewer hardships and did not want them to experience the same hardships which they faced themselves.

## Discussion

This study sought to investigate the reasons for early marriage from the perspective of 36 women who had experienced early marriage in Zahedan, Iran. The main research question was why did the participants get married early? What were their reasons? The responses of the participants were classified into three main categories: “early marriage as a transcendental coercion (extra-personal coercion)”, “early marriage as a solution or a kind of problem solving”, and “early marriage as a voluntary act”.

The concepts and categories extracted from this study offer a multidimensional picture of early marriage. Previous studies have emphasized the compulsory nature of early marriage [48–52], but our findings showed that early marriage can sometimes be regarded as a reasonable and strategic decision with personal and social functions. The categories extracted in this research are aligned with some other studies [49, 53] that point to cultural and family pressures that drive girls to early marriage. On the other hand, our result suggesting that some girls voluntarily choose to marry early after calculating the profits and losses is compatible with some other studies [54, 55]. Each of these findings is discussed in detail below.

Early marriage as a transcendental or extra-personal coercion was one of the main categories extracted in this study. This category represents the cultural, social, and geographical context that force individuals to follow its norms. The ethnic structure in Sistan and Baluchestan province and the dominance of the spirit of collectivism over individualism require individuals to conform with existing norms. These norms may have had positive social functions for the people of a specific region in the distant past, ensuring their persistence to this date, but today with the change of societies and the move toward modernity, some of these norms are defunct and have lost their constructive role. However, some of these cultural customs, including early marriage, have resisted changes. Although most of our participants stated that they were opposed to the early marriage of their children for various reasons, their opposition alone cannot prompt any significant alteration in the cultural status of this phenomenon in the patriarchal tribal system where gender discrimination prevails. Therefore, gender norms, the traditionalism of many families, and reluctance to deviate from entrenched norms reinforce the persistence of early marriage. Due to the dominance of these customs and traditional values, legal coercion is not sufficient because legal barriers and punishment can be bypassed by religious justifications, secrecy, or non-official registration of marriage and divorce, all of which further undermine women’s legal rights in this region. Grijns and Horii [56] also pointed to the contrast between tradition and law in their study in Indonesia, where people tend to stick to traditions despite improvements in the legislative system.

The other two main categories of this study included “early marriage as a solution or a kind of problem-solving” and “early marriage as a voluntary act”, which implied an informed decision based on rational profit-loss calculations to marry at a rather young age. These individuals refused being coerced into marriage and instead pointed to the factors and circumstances leading them to embrace early marriage as a solution and a voluntary act. For them, marriage was an option that could create better conditions, rescuing them from unfavorable family environment (incompatible interfamilial relationships, gender discrimination, neglect, poverty, excessive parental control, etc.) and conferring a desirable family. These factors have been confirmed in numerous studies as the causes of early marriage [57, 58]. Some of these girls might have even insisted on marrying at a young age with a person who they were interested in.

The question is whether this choice takes place with knowledge and awareness among a wide or limited number of alternatives or is the choice of early marriage a choice between bad and worse? To answer this question, it is necessary to pay attention to the position of women in the social and cultural context of Sistan and Baluchestan province, where women have poor access to sufficient information for making decisions. It should be considered if the development, growth, and awareness of girls in a given cultural context provide them with multiple choices, or they just have to choose between marriage and celibacy or late marriage.

The two concepts of “real agency” and “imaginary agency”, which were extracted from the interviews, indicated that two types of decisions can be recognized among young girls in this area. Since the cultural and social context of the region defines early marriage of girls as a social norm, some families prepare their girls for early marriage, so that these girls acquire the necessary skills for marital life at a young age. Therefore, such girls are ready to get married and even choose among the suitors based on their own priorities and wills (real agency). However, some girls choose to get married without being sufficiently prepared for a marital life and without having enough knowledge in this regard (imaginary agency). For the group characterized by imaginary agency, early marriage seems to be due to the fact that the girls are trapped in a cycle of cultural poverty: living in a dysfunctional cycle in which gender inequality, repressive social expectations, cultural violence, and gender stereotypes are perpetuated and reproduced as a traditional marital pattern. After marriage, these girls may face personal and social problems such as depression, marital conflicts, violence, poverty, etc. On the other hand, in the group showing real agency, early marriage did not entail such negative consequences, and the women were truly satisfied with their marriage, did not see it as an obstacle, and even continued their education after marriage. Therefore, considering the existence of both real and imaginary agency in adolescent females, it seems necessary to improve cultural conditions and empower families and girls on different issues, including marriage. Early marriages, if performed with lack of knowledge and by coercion, will certainly have dire short- and long-term consequences for societies. These outcomes include increased risk of miscarriage, premature birth, maternal and neonatal mortality, lost educational opportunities, lack of social development, inability to deal with complex marital life situations, and increased incidence of mental health problems and depression [59].

### Strengths, limitations and public health implications

The results of this research provide an in-depth understanding of early marriage, and they can be used by Iranian health planners to improve health programs.

This research used a qualitative method, which is incapable of exploring causal relationships. The generalizability of the findings may be limited because the interviews were conducted with females who agreed to participate in the interview (thus excluding those who were not willing to talk about their marriage experience) and all participants in the study were from Zahedan. In addition, our findings and recommendations are related to women who married between the ages of 12 and 18, and they cannot be generalized to marriages occurring under 12 years of age.

The intervention programs that are meant to reduce early marriage should consider the following observations:

1. To reduce early marriages that are forced and mostly originated in the norms of local and ethnic culture, changing laws alone is not effective and it is imperative to prioritize health interventions that are culturally-specific and tailored to the needs of teenage girls. Furthermore, efforts need to be made to smoothly and gradually reform cultural thoughts and attitudes and break the cycle of cultural poverty. Cultural changes, especially those affecting thoughts and ideas, sometimes face serious resistance. Therefore, any cultural and health program in this area should be smooth, slow, and circumspect. This goal definitely requires a long time of planning and the cooperation of locally credible NGOs that have acquired people’s trust in the region.
2. Regarding early marriages that happen without coercion and based on voluntary action, cultural authorities and health policy makers should pay attention to the “management” of early marriages. In this regard, it is necessary to empower teenage girls to choose a husband consciously and with the necessary criteria, as well as to raise their awareness about sexual health, fertility, and marital relations according to the local culture so as to prevent or manage the negative consequences of early marriage. Since choosing early marriage strategically could occasionally be owing to the tense family environment and marital conflicts of the parents, it is rewarding to invest in psychological interventions and family counseling.

## Conclusion

In general, the results of this research show that early marriages (between the ages of 13 and 18) are not always forced marriages. For some participants in this research, who had the experience of marriage between the ages of 13 and 18, early marriage was a solution or a voluntary action. In other words, women themselves tended to marry early and even pressured their families to marry (e.g., when they would fall in love). This type of marriage, which is less forced by ethnic and native culture, can be seen in both relatively disadvantaged cities such as Zahedan and more prosperous cities. Therefore, policymaking in the field of women’s health aimed to reduce early marriage or its negative consequences, requires knowledge of social and cultural contexts and considering the diversity of the target populations.

For future research, it is suggested to explore the reasons for early marriage among women who experienced it when they were less than 12 years of age. It might also be interesting to explore the status of early marriage in bigger cities with a more diverse cultural fabric. Finally, it is suggested to investigate the reasons why some men marry during adolescence as well as the reasons why some men marry teenage girls.

## Data Availability

The datasets generated and/or analyzed during the current study are not publicly available due to ethical reasons and to protect the integrity of participants, but are available from the corresponding author on reasonable request.
